# Media use and anti-pollution action: Empirical evidence from 2021 Chinese General Social Survey

**DOI:** 10.1371/journal.pone.0360300

**Published:** 2026-10-09

**Authors:** Lei Wang, Xiaowei Zhang, Xiaofang Wang, Deqing Jia

**Affiliations:** 1 School of Politics and Public Administration, Soochow University, Suzhou, People’s Republic of China; 2 Children’s Hospital of Soochow University, Soochow University, Suzhou, People’s Republic of China; 3 School of Marxism, Soochow University, Suzhou, People’s Republic of China; 4 The Fourth Affiliated Hospital of Soochow University, Soochow University, Suzhou, People’s Republic of China; Universiti Malaya, MALAYSIA

## Abstract

Environmental pollution increasingly requires public participation beyond everyday private pro-environmental behavior. Existing research has mainly examined media use in relation to daily pro-environmental behavior, but less is known about how traditional and new media shape citizens’ choices between institutional and non-institutional anti-pollution participation. Drawing on the O-S-R-O-R framework and using data from the 2021 Chinese General Social Survey, this study analyzes the relationships among media use, policy knowledge, and anti-pollution action. The results show that traditional media use is positively associated with institutional anti-pollution action, and this relationship is fully mediated by policy knowledge. New media use is positively associated with non-institutional anti-pollution action, but policy knowledge does not mediate this relationship. Heterogeneity analysis further shows that the effects of media use vary across age, education, and social trust groups. We highlight the importance of traditional media in supporting rational, stable engagement and suggest that stronger regulation of new media is needed, thereby reducing protest activities and safeguarding social stability.

## Introduction

Environmental pollution has become a persistent public problem that cannot be addressed solely through private pro-environmental behavior. Its consequences are not limited to ecological degradation, but extend to public health and economic stability. Research has found that exposure to pollutants has been associated with higher mortality [1]. Pollution may also undermine economic stability, as heavily harmful environmental conditions can reduce longevity and contribute to aggregate instability [2]. Although everyday pro-environmental practices are important, many pollution problems involve collective risks and conflicts over public goods. In such cases, citizens may move beyond private environmental behavior and engage in anti-pollution action. These actions can be distinguished into institutional anti-pollution actions, which are undertaken through formal and legally recognized channels, and non-institutional anti-pollution actions, which involve more contentious forms of participation outside formal institutional channels.

China provides a particularly important context for exploring these two forms of anti-pollution action. Decades of rapid industrialization have made environmental pollution one of the country’s most pressing public concerns. In response, the Chinese government has established multiple institutional channels through which citizens can report pollution incidents and participate in environmental governance. These channels provide citizens with lawful and relatively orderly ways to express environmental grievances. However, when pollution problems remain unresolved or institutional responses are perceived as ineffective, some citizens turn to non-institutional forms of participation. For example, the anti-PX protests in several Chinese cities demonstrated how environmental grievances could escalate from institutional demands to public demonstrations, attracting widespread public attention but also posing challenges to local governance [3].

Media play a huge role in this process because they are the main channels through which citizens interpret environmental risks and learn about available participation opportunities. Media-event research has shown that traditional media and social media can generate different narratives and forms of public participation around the same public issue [4]. Traditional media, including newspapers, radio and television, usually provide more official and confirmed information. New media provide faster and more interactive information exchange. Research has found that the rise of new media increased the speed and reach of public communication [5], while also increasing the risks of fragmented information [6], group polarization [7] and information cocoon [8]. Such information environments may influence citizens’ perceptions of environmental issues and their trust in institutions [9]. Consequently, traditional and new media may encourage different patterns of anti-pollution participation. Yet existing research has not sufficiently examined whether different media sources are associated with institutional and non-institutional anti-pollution action.

In this paper, we examine how traditional media use and new media use influence citizens’ anti-pollution action in China. Drawing on the O-S-R-O-R framework, we conceptualize media use as communication stimuli, policy knowledge as a cognitive mechanism, and anti-pollution action as the behavioral response. Using data from the 2021 Chinese General Social Survey, we investigate whether traditional and new media use are differently associated with institutional and non-institutional anti-pollution action, and whether policy knowledge mediates these relationships.

Our contribution to literature is threefold. First, this study focuses on anti-pollution action. Specifically, we distinguish anti-pollution action from general pro-environmental behavior and further differentiate it into institutional and non-institutional forms as two modes of public environmental participation. Unlike routine pro-environmental behaviors, which typically are private and daily actions [10–12], anti-pollution actions involve higher participation costs and more direct engagement in environmental governance. It makes actions more consequential for environmental governance and broader societal change.

Second, this study advances the literature on media and environmental participation by differentiating the effects of traditional and new media and uncovering the cognitive mechanism through which these effects occur. First, it distinguishes traditional media from new media rather than treating media use as a homogeneous construct. Traditional media mainly disseminate authoritative information, whereas new media provide a more interactive communication environment. This distinction offers an understanding of why different media types promote different forms of anti-pollution action. Second, the study identifies policy knowledge as the cognitive mechanism linking media use to anti-pollution action. By incorporating policy knowledge into the explanation, it moves beyond direct media effect and clarifies how traditional media facilitate institutional forms of public environmental participation through knowledge acquisition.

Third, this study extends the application of the Orientation–Stimulus–Reasoning–Orientation–Response model to the context of environmental behavior. Originally developed in communication research to explain how media exposure shapes behavioral responses through cognitive mechanisms [13], the O-S-R-O-R model has been applied to general political participation [13,14]. By applying this framework to anti-pollution action, this study provides new evidence on explaining how different media environments influence environmental participation through policy knowledge, extending the applicability of the model to the domain of environmental governance.

## Theoretical background and hypotheses

### Theoretical framework

We draw on the O-S-R-O-R (Orientation–Stimulus–Reasoning–Orientation–Response) model proposed by Cho, Shah [13] to develop the theoretical framework as presented in Fig 1. It provides a framework for explaining how individuals process external information from communication and translate it into behavioral responses. The model does not assume that external information directly leads to behavioral change. Instead, it proposes that information exposure influences behavior through cognitive processes.

**Fig 1:**
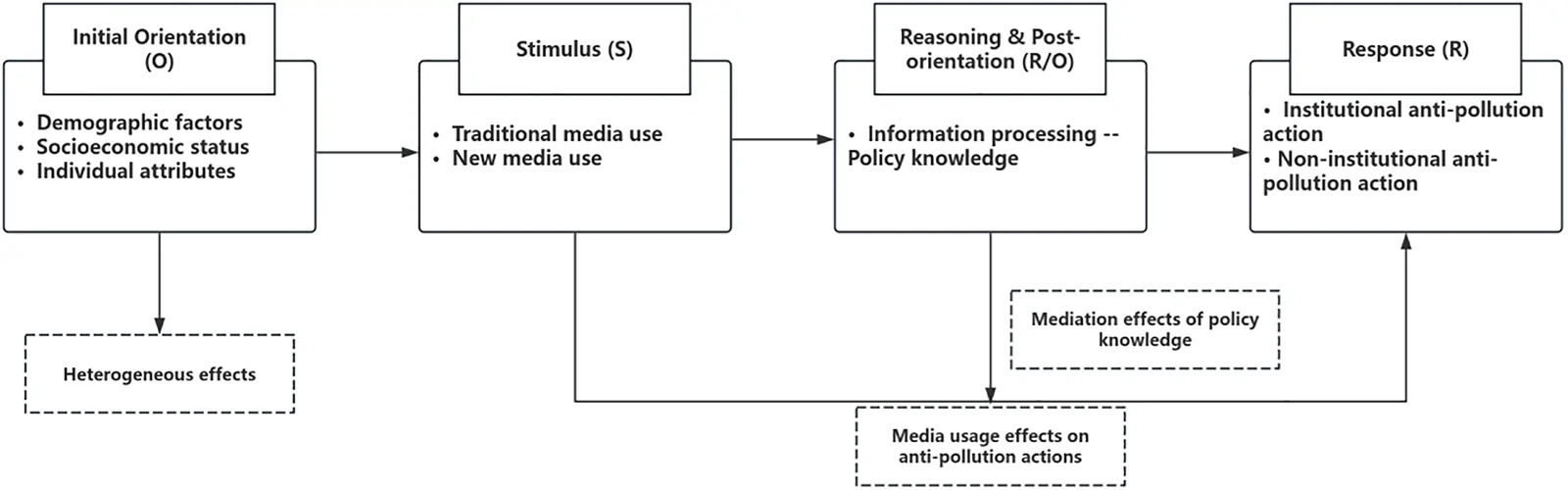
Theoretical framework.

The framework consists of several components. The first component is initial orientation (O), which reflects stable individual characteristics, such as demographic and socioeconomic backgrounds, that affect how people interpret information. The second component is stimulus (S), referring to external information sources, such as media exposure, that provide individuals with environmental information. The third component involves reasoning and post-orientation (R/O), through which individuals process the information they receive, leading to updated knowledge. Finally, these cognitive changes are translated into the response (R), which is individuals’ behavioral decisions and actions.

The O-S-R-O-R framework is mainly used in the field of political participation. For example, Cho, Shah [13] combining campaign advertising records with panel survey data on media use and campaign participation during the 2000 and 2004 U.S. election cycles, employed structural equation modeling and within the O-S-R-O-R framework they found cognitive reflection mediating the effects of campaign exposure and news consumption on political participation. Similarly, Chan [14] analyzed a representative survey of Hong Kong residents and showed that political expression and political efficacy mediate the effects of social media use on participation.

Building on this theoretical perspective, we extend the application of the O-S-R-O-R framework to anti-pollution action. Specifically, we examine how different media sources affect anti-pollution action through policy knowledge. In our study, traditional media use and new media use are conceptualized as two different communication stimuli, and policy knowledge is examined as the cognitive mechanism linking media exposure to anti-pollution action. Anti-pollution action is further differentiated into institutional and non-institutional ones, allowing us to assess whether different media environments promote different modes of participation. In addition, following the initial orientation component of the O-S-R-O-R framework, this study also investigates whether the effects of media use vary across demographic factors.

### Literature review

Previous research has extensively examined the determinants of pro-environmental behavior, highlighting the importance of different psychological and socio-demographic factors (e.g., Vicente-Molina, Fernández-Sainz [10], Otto and Kaiser [11], Varela-Candamio, Novo-Corti [12], Berenguer, Corraliza [15], Otto, Neaman [16], Miller, Rice [17] and Moon [18]). For example, gender plays an important role. Vicente-Molina, Fernández-Sainz [10] surveyed 1,089 Spanish university students through quota sampling to analyze pro-environmental behaviors (recycling, public transit, green consumption). Using gender-disaggregated ordered logit models, they found that female students with a scientific background and positive environmental attitudes were the most likely to engage in these daily pro-environmental actions. Regarding age, a positive relationship with ecological behavior has been identified. Otto and Kaiser [11] drew on a sample of 779 adults from Saxony-Anhalt, Germany and environmental behavior was measured using the General Ecological Behavior scale, which captures a broad range of everyday pro-environmental behaviors, such as reusing shopping bags and membership in environmental organizations. They found that older individuals were more likely to engage in ecological behaviors than younger individuals. Varela-Candamio, Novo-Corti [12] analyzed questionnaire data from 117 residents in Spain using structural equation modeling. Measuring green behavior through a range of everyday pro-environmental activities, they found that environmental education was the most important driver of green behavior, outperforming motivational and interpersonal factors. Miller, Rice [17] measured pro-environmental behavior using both the frequency of everyday environmental practices, such as recycling and using reusable shopping bags and participation in public environmental actions, such as donating to environmental causes and signing environmental petitions. With survey data from 11 countries, their findings show that environmental attitudes are a strong predictor of pro-environmental behaviors.

Although previous studies have explored a wide range of factors, they mainly focused on everyday pro-environmental behaviors, with little attention given to anti-pollution action. Unlike routine green behaviors, anti-pollution actions are directed toward addressing severe environmental degradation and often involve collective engagement with environmental governance rather than individual lifestyle changes. Such actions typically carry higher participation costs and potential conflicts with governmental or industrial actors [19]. This study focuses specifically on anti-pollution actions and further distinguishes between institutional and non-institutional forms of participation. By differentiating these two participation channels, we extend the literature beyond everyday pro-environmental behavior and provide an understanding of citizens’ engagement in environmental governance.

The relationship between media use and environmental behavior has also attracted increasing scholarly attention [20–22]. However, compared with the evidence on the direct association between media use and environmental behavior, research on the mechanisms through which media exposure is translated into action remains limited. As exceptions, some studies identified several cognitive and psychological mechanisms. For example, with a nationwide survey of 1,074 adults in Taiwan and structural equation modeling, Huang [22] provides evidence that environmental beliefs and environmental self-efficacy mediated the relationship between media use and pro-environmental behavior. Liu, Han [23] analyzed data from 9,473 Chinese respondents employing structural equation modeling. Their findings showed that Internet use influenced pro-environmental behavior both directly and indirectly through mediators such as environmental knowledge and perceived environmental pollution threats. Li, Wang [24] further showed that environmental concern and environmental locus of control mediated the relationship between media exposure and plastic-reduction behaviors. Shao and Yu [25] combined a preregistered experiment (N = 284) and a cross-sectional survey (N = 507) of Chinese youths and found that media exposure increased eco-anxiety and pro-environmental behavior, with eco-anxiety serving as a mediator between media exposure and behavioral engagement. Shen, Liang [26] further explored the mechanisms through which social media–based green communities affect pro-environmental behavior. With survey data from 585 respondents in Pakistan, they found that browsing green communities was positively associated with pro-environmental behavior through the sequential mediating effects of participation in green communities and environmental concern. Novitaningtyas and Paramita [27] examined young tourists in Indonesia and found that perceived authenticity on social media increased pro-environmental behavior through emotional satisfaction and environmental self-efficacy.

Although these studies have advanced understanding of the cognitive mediation linking media exposure to environmental behavior, much less attention has been paid to policy knowledge. Different from environmental cognitive factors, policy knowledge reflects citizens’ understanding of policies. Such knowledge provides citizens with the information needed to evaluate policy performance, identify appropriate institutions, and choose effective channels for public participation. This is particularly important for anti-pollution actions, which often involve direct engagement with environmental governance rather than simple daily environmental behaviors.

### Hypotheses

Considering that traditional media in China are mainly official media, they bear significant social responsibilities, which have established a high level of authority [28]. Traditional media place strong emphasis on factual accuracy and objectivity, avoiding exaggeration or distortion of information [29]. Given their objective and rational reporting style, audiences who rely more on traditional media are likely to approach environmental issues in a more reasoned manner and seek solutions within the legal framework [30]. Thus, we propose the following hypotheses:

H1: Individuals who use traditional media more often are more inclined to engage in institutional environmental actions.

The fragmented and emotionally charged nature of new media environments often increases users’ susceptibility to impulsive reactions [31]. Gong, Lu [32] showed that online discussion can amplify contestation and make public issues more emotionally and normatively charged. In such environments, individuals are more easily influenced by opinion leaders [33], whose provocative statements can intensify collective emotions and fuel group polarization. Furthermore, algorithmic reinforcement of user preferences leads to the formation of information cocoons, limiting exposure to diverse viewpoints [8]. As a result, individuals are increasingly likely to engage in non-institutional forms, driven by emotional intensity rather than by rational deliberation.

Such effect on non-institutional is already found in previous findings. For example, Facebook groups played a role in organizing the 2018 educator walkouts in Oklahoma and Arizona, which were large-scale, statewide actions [34]. In environmental fields, Borch, Munk [35] reveals that Facebook serves as a resonance space for advocating anti-wind power viewpoints. Similarly Chia [36] finds that youth climate activists in Canada used social media as a tool to mobilize the global climate strike movement. Thus, we propose the following hypotheses:

H2: Individuals who use new media more often are more likely to engage in non-institutional environmental actions.

## Data and empirical strategy

### Data

In this paper, we use the 2021 Chinese General Social Survey (CGSS) for analysis. The CGSS, conducted by Renmin University of China, adopts a multi-stage stratified probability sampling strategy to ensure representativeness. Specifically, the 2021 CGSS covers 85 cities across 30 provinces in mainland China and includes 8,148 respondents. Structured questionnaires were employed to collect data. The information gathered includes residents’ engagement in both institutional and non-institutional anti-pollution behaviors, their use of traditional and new media, and their socio-demographic characteristics. For the purposes of this study, we focused on observations characterized by high perceived environmental risk, based on individuals’ assessments of the severity of their local environmental conditions (Individuals with high environmental risks usually are more sensitive to environmental information presented on media, which helps us clearly observe the effect of media exposure, minimizing the potential noise that could obscure this relationship in the general population. Based on respondents’ answers to the environmental risk perception section presented in S1 Table, those with scores above the median of the full sample are classified as high environmental risk individuals.).

To construct our sample, we first retained respondents with valid information on environmental risk perception and restricted the sample to those with a high-risk perception. This produced a high-risk subsample of 5,303 respondents. We then constructed two dependent variables: institutional anti-pollution action and non-institutional anti-pollution action. After excluding missing responses on the outcome variables, the final analytical sample contained 897 observations. The detailed measurement of environmental risk is provided in S1 Table.

### Empirical strategy

We set our empirical model as follows based on a Probit model:


$$\begin{array}{c} {Inst\_behavior}_{i}=\alpha_{\text{1}}+\alpha_{\text{2}}{Trad\_media}_{i}+{\alpha_{\text{3}}{New\_media}_{i}+\alpha }_{\text{4}}X_{i}+\varepsilon_{i} \end{array}$$ (1)

$$\begin{array}{c} {Noninst\_behavior}_{i}=\beta_{\text{1}}+\beta_{\text{2}}{Trad\_media}_{i}+\beta_{\text{3}}{New\_media}_{i}+\beta_{\text{4}}X_{i}+\varepsilon_{i} \end{array}$$ (2)

In Equation (1), ${Inst\_behavior}_{i}$ is defined as a binary variable which indicates whether the respondent *i* participated in an institutional anti-pollution action, i.e., signing a petition regarding an environmental pollution issue. If the respondent *i* participated before, then the value is assigned as one, otherwise zero. In the Equation (2), ${Noninst\_behavior}_{i}$ is a binary variable taking the value of 1 if the respondent *i* engaged in a non-institutional anti-pollution action, i.e., participated in a protest or demonstration and 0 otherwise.

${Trad\_media}_{i}$is a variable indicating the frequency with which respondent *i* uses traditional media, calculated as the sum of scores across newspapers, magazines, radio, and television. ${New\_media}_{i}$ represents the frequency of new media usage by respondent *i*, obtained by summing scores for the Internet and mobile subscription services. As detailed in Table 1, media usage frequency is measured using a five-point Likert scale, where 1 is never use and 5 is very frequent use.

**Table 1:** Media use items.

Variable	Description	Definition
Traditional media	Newspapers	In the past year, how often did you use the following media: 1 = Never, 2 = Rarely, 3 = Occasionally, 4 = Frequently, 5 = Very frequently
	Magazines	
	Radio	
	Television	
New media	Internet (including mobile internet access)	
	Mobile subscription messages	

Notes: Authors’ design.

Given that ${Trad\_media}_{i}$ and ${New\_media}_{i}$ are constructed from different numbers of measurement items, we then use a normalization approach to these two variables in order to address scale differences. The specific normalization process is detailed below:


$$\begin{array}{c} {Trad\_media\_norm}_{i}={[Trad\_media}_{i}-\text{ min}({Trad\_media}_{i})]/[\text{max}\left({Trad\_media}_{i}\right)-\text{ min}({Trad\_media}_{i})] \end{array}$$ (3)

$$\begin{array}{c} {New\_media\_norm}_{i}={[New\_media}_{i}-\text{ min}({New\_media}_{i})]/[\text{max}\left({New\_media}_{i}\right)-\text{ min}({New\_media}_{i})] \end{array}$$ (4)

Furthermore, $X_{i}$ includes the control variables shown in Table 2, which are considered potential factors affecting individual environmental behaviors. $\alpha_{\text{2}}$, $\alpha_{\text{3}},\;\beta_{\text{2}}\;$and $\beta_{\text{3}}$ are estimated to capture the effects of tradition media usage and new media usage on individual anti-pollution actions. Finally, $\varepsilon_{i}$ represents the random error term.

**Table 2:** Variable descriptions.

Variable	Description	Mean	SD	Min	Max
Inst_behavior	1 = Participated in signing a petition regarding an environmental pollution issue, 0 = Otherwise	0.030	–	0	1
Noninst_behavior	1 = Participated in a protest or demonstration for an environmental pollution issue, 0 = Otherwise	0.010	–	0	1
Trad_media_norm	Normalized traditional media usage frequency	0.280	–	0	1
New_media_norm	Normalized new media usage frequency	0.420	–	0	1
Age	Respondent’s age	55.810	17.080	22	99
Male	1 = Male, 0 = Female	0.460	–	0	1
Rural	1 = Rural, 0 = Otherwise	0.600	–	0	1
Marital Status	1 = Married, 0 = Otherwise	0.730	–	0	1
Highest Education level	0 = No education, 1 = Primary school education, 2 = Junior high school education, 3 = Senior high school, 4 = Undergraduate, 5 = Graduate and above	2.170	1.290	0	5
Employment	1 = Employed, 0 = Unemployed	0.360	–	0	1
Income	Total yearly income (CNY)	41,754	270000	0	1.00E + 07
Majority	1 = Han majority, 0 = Otherwise	0.930	–	0	1
Election	1 = Election voting participated, 0 = Otherwise	0.530	–	0	1
Social trust	Do you agree: Most people in society are trustworthy, measured on a 5-point scale from strongly not agree to strongly agree.	3.670	0.980	1	5
Social interaction	The frequency of engagement in social networking during free time, measured on a 5-point scale from not at all to very frequent.	2.640	1.110	1	5

Notes: Authors’ calculations. The number of observations for Inst_behavior and Noninst_behavior is 897 due to missing records. All other variables have 5,303 observations.

## Results

### Baseline results

Table 3 reports the results, where the reported coefficients are replaced by marginal effects. In Model 1, traditional media usage is found to significantly increase the likelihood of engaging in institutional anti-pollution actions at the significant level of 1%. Specifically, a one-unit increase in traditional media usage is associated with a 9.4 percentage point higher probability of participating in actions such as signing an environmental petition.

**Table 3:** Media usage and anti-pollution actions.

	Model 1	Model 2
Variables	Inst_action	Noninst_action
Trad_media_norm	0.094***	−0.060*
	(0.036)	(0.035)
New_media_norm	0.034	0.022*
	(0.023)	(0.013)
Control variables	Yes	Yes
Observations	897	540
Pseudo-R^2^	0.076	0.279

Notes: Authors’ computation. Robust standard errors are reported in parentheses. *** indicates significance levels of 1%. * indicates significance levels of 10%. Weighted estimates of the baseline regressions are reported in S3 Table as a robustness check.

In Model 2, the results indicate that greater traditional media usage reduces the likelihood of non-institutional anti-pollution actions, such as participating in protests or demonstrations, by 6 percentage points. However, increased new media usage is associated with a 2.2 percentage point increase in the probability of engaging in non-institutional actions. This effect is statistically significant at the 1% level.

To address potential endogeneity from reverse causality and omitted variables, we further conduct an IV-Probit estimation, as reported in Table 5. As the instrumental variable, we use the average traditional and new media usage of other respondents within the same city, which is IV_Trad_media and IV_New_media presented in Table 4. This city-level mean is expected to influence an individual’s own media usage but is unlikely to directly affect their anti-pollution actions, thereby satisfying the relevance and exclusion criteria for a valid instrument. The use of averages from other respondents as instruments has been widely adopted in the literature to mitigate endogeneity concerns [37–39]. As shown in Table 4, the selected instrumental variables exhibit significant effect on the endogenous variables and the first-stage F-test statistic exceeds the threshold of 10, suggesting that weak instrument concerns are unlikely and the instrument is valid [40].

**Table 4:** First-stage regression results for instrumental variables.

	Model 1	Model 2
Variables	Trad_media_norm	New_media_norm
IV_Trad_media	0.479***	
	(0.059)	
IV_New_media		0.371***
		(0.039)
Control variables	Yes	Yes
Observations	5291	5291
R^2^	0.174	0.412
F-test	80.59	424.08

Notes: Authors’ computation. Robust standard errors are reported in parentheses. *** indicates significance levels of 1%. Given that both Trad_media_norm and New_media_norm are continuous variables bounded between 0 and 1, we employ ordinary least squares (OLS) regression for estimation.

The results of Table 5 show that, after accounting for endogeneity, the main findings remain consistent with previous estimates and support our hypotheses regarding the positive effect of traditional media usage on institutional anti-pollution actions and the negative effect of new media usage on non-institutional actions. The difference is that the effect of traditional media usage on non-institutional actions in Model 2 of Table 5 becomes statistically insignificant compared to Table 3. Moreover, the estimated coefficient in Table 5 is larger than that in Table 3. A possible explanation is that anti-pollution actions may in turn influence media usage, leading to a negative correlation between media usage and the error term. As a result, the Probit model may underestimate the effect of media usage on anti-pollution actions.

**Table 5:** Robustness for media usage and anti-pollution actions.

	Model 1	Model 2
Variables	Inst_action	Noninst_action
Trad_media_norm	5.499***	2.740
	(1.459)	(3.467)
New_media_norm	−0.280	3.600*
	(2.555)	(1.912)
Control variables	Yes	Yes
Observations	897	540

Notes: Authors’ computation. Robust standard errors are reported in parentheses. *** indicates significance levels of 1%. * indicates significance levels of 10%. The marginal coefficients are presented in the table.

### Channel analysis

Previous research has found that media use can significantly influence political knowledge [41] and then promote political participation [42,43]. Based on this, we propose that exposure to political knowledge through media, specifically knowledge of environmental policies and their implementation, may enhance individuals’ willingness to take action against pollution. To capture this mechanism, we construct a policy knowledge index based on respondents’ self-reported understanding of 13 major environmental policies, including ecological civilization, ecological compensation, pollution prevention and control, environmental inspection, and public participation in environmental protection. Respondents rated their understanding of each policy on a four-point scale ranging from 0 (Do not understand) to 3 (Understand very well), and the overall policy knowledge index was calculated as the sum of these scores. Detailed information on the measurement is provided in S2 Table.

As Table 5 indicates, traditional media usage significantly influences institutional actions, while new media usage is associated only with non-institutional actions. Thus, in Table 6 and Table 7, we further examine the mediation role of policy knowledge within these two models. We use the IV-Bootstrap method, drawing 1,000 repeated random samples and constructing 95% confidence intervals. The results based on the bootstrap estimation are presented in Table 6 and Table 7.

**Table 6:** Mediation effects of policy familiarity between traditional media use and institutional anti-pollution actions.

Path	Inst_action			
Indirect effect	Coeff.	S.E.	LLCI	ULCI
	0.087**	0.04	0.008	0.166
Direct effect	Coeff.	S.E.	LLCI	ULCI
	0.188	0.205	−0.214	0.591

Notes: Authors’ own computation. LLCI and ULCI indicate the lower and upper limit of the confidence intervals; ** indicates a significant level of 5%. Policy knowledge: calculated by summing the values from the 14 environmental policies, each rated on a 4-point scale, which is presented in S2 Table.

**Table 7:** Mediation effects of policy familiarity between new media use and non-institutional anti-pollution actions.

Path	Noninst_action			
Indirect effect	Coeff.	S.E.	LLCI	ULCI
	0.010	0.010	−0.009	0.029
Direct effect	Coeff.	S.E.	LLCI	ULCI
	−0.025	0.025	−0.074	0.023

Notes: Authors’ own computation. LLCI and ULCI indicate the lower and upper limit of the confidence intervals. Policy knowledge: calculated by summing the values from the 14 environmental policies, each rated on a 4-point scale, which is presented in S2 Table.

Table 6 shows that policy knowledge exerts a significant positive indirect effect on institutional actions, whereas the direct effect of traditional media usage becomes non-significant when controlling for policy knowledge. This pattern suggests that policy knowledge serves as a full mediator in the relationship between traditional media usage and institutional anti-pollution actions. However, in Table 7, no mediating effect of policy knowledge is observed for non-institutional actions in the new media model.

### Heterogeneity analysis

The impact of media may vary across different populations, leading to varying effects among different demographic groups. In this paper, we propose that age, education level, and social trust serve as potential moderators of media influence.

We propose that older adults may be more susceptible to media influences and then engage in more anti-pollution behaviors. This could result from several factors: first, older individuals tend to exhibit more sensitivity to environmental issues due to concerns about their health [44]. Second, given their familial responsibilities [45], they may also demonstrate increased environmental responsiveness in order to protect their families. From empirical evidence, previous research has shown that older individuals appear more inclined to minimize environmental harm, and engage in more pro-environmental activities [11,46]. To confirm this expectation, we include an interaction term between different media usages and age in Eq. (1) and (2), as shown in Table 8. We find that the interaction term is positive in Model 2, indicating that an increase in respondent’s age strengthens individual intention to engage in non-institutional behaviors.

**Table 8:** The heterogeneous effects of media usage on anti-pollution actions.

	Model 1	Model 2	Model 3	Model 4	Model 5	Model 6
Variables	Inst_action	Noninst_action	Inst_action	Noninst_action	Inst_action	Noninst_action
Trad_media_norm * Age	0.015	0.148*				
	(0.024)	(0.089)				
New_media_norm * Age	−0.028	0.116***				
	(0.018)	(0.032)				
Trad_media_norm * Education			0.243	0.943		
			(0.384)	(1.174)		
New_media_norm * Education			0.149	−0.952*		
			(0.260)	(0.488)		
Trad_media_norm * Social trust					0.091	−0.130
					(0.474)	(1.228)
New_media_norm * Social trust					−0.685**	−0.836**
					(0.317)	(0.401)
Control variables	Yes	Yes	Yes	Yes	Yes	Yes
Observations	897	540	897	540	897	540
Pseudo-R^2^	0.086	0.333	0.079	0.305	0.093	0.302

Notes: Authors’ computation. Robust standard errors are reported in parentheses. *** indicates significance levels of 1%. ** indicates significance levels of 5%. * indicates significance levels of 10%.

Education level may moderate media effects. We propose that higher levels of education alleviate the influence of media on the tendency to engage in non-institutional anti-pollution actions. Individuals with higher education typically possess greater environmental knowledge and cognitive resources, which foster more rational responses and help reduce radicalized behaviors [47]. Their broader knowledge base may also help them resist the echo chamber effects created by media exposure [8]. Applying a similar approach as before, we observe a negative interaction between *New_media_norm* and *Education* on non-institutional actions (see Model 4 of Table 8), suggesting that the positive effect of new media on non-institutional actions is weakened at higher levels of education.

Moreover, we expect that social trust weakens the positive effects of media usage on anti-pollution actions. Individuals with higher levels of social trust tend to have greater confidence in government and society, believing that existing systems are capable of effectively addressing environmental issues. Thus, when exposed to media reports on the Internet, they are less likely to be influenced by conflicting or sensational information. In this case, they are less motivated to engage in anti-pollution actions. From empirical evidence, Australia’s potable reuse schemes study shows that social trust mitigates perceived health risks and enhances public acceptance of recycled water [48]. Based on survey data from 35 countries, Smith and Mayer [49] find that insufficient trust diminishes the translation of risk perceptions into policy support. Table 8 shows that, in line with our expectations, social trust significantly weakens the influence of new media on anti-pollution actions.

## Discussion

This study examined how media use shapes citizens’ engagement in anti-pollution action. By distinguishing between traditional media and new media, and between institutional and non-institutional anti-pollution action, we show that different media environments are associated with different modes of public environmental participation. The results indicate that more frequent use of traditional media increases the probability of participating in institutional anti-pollution action, such as signing environmental petitions, by 9.4 percentage points. By contrast, more frequent use of new media increases the probability of participating in non-institutional anti-pollution action by 2.2 percentage points. These findings remain robust in the IV-Probit estimation and are in line with our hypothesis.

These findings extend previous research on media use and environmental behavior by focusing on anti-pollution actions. Previous studies have shown that media use can promote environmental behavior [10,12,17], but they have mainly examined everyday environmental practices or broad pro-environmental behavior. Our study finds that media usage enhances participation in anti-pollution actions, but compared to traditional media, new media is more likely to trigger radical pro-environmental actions. Specifically, traditional media such as newspapers, magazines, and television, which are largely controlled by official institutions, serve as reliable sources of legal knowledge and policy interpretation [28,29]. Exposure to such authoritative information enables individuals to develop a deeper understanding of legal norms. As a result, individuals are more likely to regulate their actions to avoid legal violations [30]. However, new media platforms such as the internet which is characterized by an abundance of mixed-quality information, making individuals highly susceptible to the influence of opinion leaders [33]. This can intensify collective emotions and exacerbate group polarization. Moreover, the formation of information cocoons, which limit exposure to diverse perspectives, further contributes to the escalation of extreme behaviors [8].

The mediation analysis further clarifies the cognitive mechanism linking media use to anti-pollution action. We find that policy knowledge fully mediates the relationship between traditional media use and institutional anti-pollution action. This result is consistent with previous research showing that knowledge can serve as an important cognitive mechanism between media exposure and pro-environmental behavior. For instance, Liu, Han [23] found that Internet use promoted pro-environmental behavior through environmental knowledge. Different from this study, we show that the relevant cognitive mechanism in anti-pollution action is not only environmental knowledge, but also policy knowledge. Traditional media may promote institutional participation as they provide credible and policy-oriented information that helps citizens understand how environmental governance works and which formal channels are available. However, no significant mediation role is found in new media model.

Regarding heterogeneity, we find some individual characteristics have moderating effects on the role of the media usage on anti-pollution actions. Specifically, older individuals are more responsive to media reports and are more likely to be influenced to engage in anti-pollution behaviors. This may be explained by their greater concern for health [44] and stronger sense of familial responsibility [45], which enhance their motivation to pursue a better environment. Education can offset the effect of new media by promoting more rational decision-making and then reducing individuals’ tendency to engage in radical behaviors [8]. Furthermore, social trust also helps mitigate the impact of new media exerted in anti-pollution actions. Individuals with higher levels of social trust tend to have greater confidence in governmental and societal institutions, believing that existing systems are capable of effectively addressing environmental issues [49], which in turn reduces their likelihood of engaging in anti-pollution actions.

## Conclusion

This study examined how different media environments shape citizens’ anti-pollution action in China. The study shows that media use does not influence environmental participation in the same way. Traditional media use is more strongly associated with institutional anti-pollution action, while new media use is more strongly associated with non-institutional anti-pollution action. The mediation analysis further shows that policy knowledge fully mediates the relationship between traditional media use and institutional action.

Overall, this study makes several contributions. First, it shifts attention from general pro-environmental behavior to anti-pollution action as a more specific and governance-oriented form of public environmental participation, and further distinguishes between institutional and non-institutional action. Second, by applying the O-S-R-O-R framework, the study extends it to the context of environmental behavior and identifies policy knowledge as a cognitive mediation.

Our results have some policy implications. Since traditional media appear to promote institutional anti-pollution action through policy knowledge, public agencies could make use of traditional media to explain environmental regulations and citizens’ rights in environmental governance. Such communication should focus not only on reporting pollution problems, but also on clarifying how citizens can seek formal and lawful solutions. At the same time, the association between new media use and non-institutional action suggests the need to improve the quality of online environmental communication. Rather than simply restricting new media, policymakers should strengthen timely information disclosure and digital literacy training, especially in relation to group polarization and information cocoons. The heterogeneity results further indicate that higher levels of education and social trust are associated with a lower likelihood of engaging in non-institutional action. These findings imply that strengthening environmental policy education and enhancing the transparency and responsiveness of environmental governance may encourage citizens to channel environmental grievances through institutional ways.

This study may bear several limitations. First, the use of cross-sectional data limits our ability to conduct dynamic analysis and may raise concerns about endogeneity. Although we attempt to address this issue using an IV-Probit approach, future research could employ panel data to further verify the robustness of our findings. Second, due to data constraints, we are unable to examine additional mediating mechanisms underlying the relationship between new media usage and non-institutional actions. Investigating such mediators in future studies would provide a more comprehensive understanding of the links between media exposure and environmental behaviors.

## Supporting information

S1 Table Measurement of environmental risk perception. (DOCX)

S2 Table Measurement of policy knowledge. (DOCX)

S3 Table Robustness of media usage and anti-pollution actions. (DOCX)
