# Supplementary material for: Media use and anti-pollution action: Empirical evidence from 2021 Chinese General Social Survey
Source: PLoS One. 2026 Oct 9;21(10):e0360300. doi: 10.1371/journal.pone.0360300 (PMC13652217; doi:10.1371/journal.pone.0360300)
Supplement: Supplementary file 1 [file pone.0360300.s001.docx]

**S1 Table. Measurement of environmental risk perception**

| Variable | Description | Definition |
| --- | --- | --- |
| Environmental risk | Air pollution | On each item, how severe do you think they are in your residential area?  0 = No such problem, 1 = Not severe, 2 = Not very severe, 3 = Moderate, 4 = Relatively severe, 5 = Very severe |
|  | Water pollution |  |
|  | Soil pollution |  |
|  | Noise pollution |  |
|  | Industrial waste pollution |  |
|  | Household waste pollution |  |
|  | Building and construction waste pollution |  |
|  | Forest vegetation destruction |  |
|  | Biodiversity loss (e.g., decline of wild species |  |
|  | Lack of green space |  |
|  | Freshwater resource shortage |  |
|  | Land desertification |  |
|  | Soil erosion |  |
|  | Resource wastage |  |
|  | Extreme weather events |  |
|  | Food safety |  |
| Notes: Authors’ design. The environmental risk variable is defined as the total sum of the severity scores assigned by each respondent to all environmental issues. Respondents with scores above the median of the full sample are classified as high environmental risk individuals. | | |
