# Supplementary material for: Media use and anti-pollution action: Empirical evidence from 2021 Chinese General Social Survey
Source: PLoS One. 2026 Oct 9;21(10):e0360300. doi: 10.1371/journal.pone.0360300 (PMC13652217; doi:10.1371/journal.pone.0360300)
Supplement: Supplementary file 2 [file pone.0360300.s002.docx]

**S2 Table. Measurement of policy knowledge**

| Variable | Description | Definition |
| --- | --- | --- |
| Policy knowledge | Ecological civilization policy | How well do you know about each policy?  0 = Do not understand, 1 = Understand a little, 2 = Understand fairly well, 3 = Understand very well, |
|  | Ecological compensation policy |  |
|  | Ecological conservation redline policy |  |
|  | Ecological civilization system reform |  |
|  | National ecological civilization pilot zones |  |
|  | Target evaluation system for ecological civilization construction |  |
|  | National main functional zoning plan |  |
|  | Circular economy promotion policy |  |
|  | Environmental inspection and patrol policy |  |
|  | Air pollution prevention and control action plan |  |
|  | Water pollution prevention and control action plan |  |
|  | Soil pollution prevention and control action plan |  |
|  | Public participation regulations for environmental protection |  |
|  | Accountability system for ecological and environmental damage under party leadership |  |
| Notes: Authors’ design. The policy knowledge variable is defined as the total sum of the understanding scores assigned by each respondent across all listed environmental policies. | | |
