# Supplementary material for: Media use and anti-pollution action: Empirical evidence from 2021 Chinese General Social Survey
Source: PLoS One. 2026 Oct 9;21(10):e0360300. doi: 10.1371/journal.pone.0360300 (PMC13652217; doi:10.1371/journal.pone.0360300)
Supplement: Supplementary file 3 [file pone.0360300.s003.docx]

**S3 Table. Robustness of media usage and anti-pollution actions**

|  | Model 1 | Model 2 |
| --- | --- | --- |
| Variables | Inst_action | Noninst_action |
| Trad_media_norm | 1.022* | -3.418*** |
|  | (0.579) | (1.282) |
| New_media_norm | 0.596 | 0.917* |
|  | (0.380) | (0.475) |
| Control variables | Yes | Yes |
| Observations | 897 | 540 |
| Pseudo-R^2^ | 0.058 | 0.297 |
| Notes: Authors’ computation. Robust standard errors are reported in parentheses. This table reports the weighted estimates of the baseline regressions (Table 3) using the CGSS sampling weights. *** indicates significance levels of 1%. * indicates significance levels of 10%. | | |
